# Transcriptome profiling of *longissimus thoracis* muscles identifies highly connected differentially expressed genes in meat type sheep of India

**DOI:** 10.1371/journal.pone.0217461

**Published:** 2019-06-06

**Authors:** Reena Arora, Naveen Kumar S., Sudarshan S., Mohamed Nadeem Fairoze, Mandeep Kaur, Anju Sharma, Yashila Girdhar, Sreesujatha R. M., Suresh K. Devatkal, Sonika Ahlawat, Ramesh Kumar Vijh, Manjunatha S. S.

**Affiliations:** 1 ICAR-National Bureau of Animal Genetic Resources, Karnal, Haryana, India; 2 Karnataka Veterinary Animal and Fisheries Sciences University, Bangalore, Karnataka, India; 3 ICAR-National Research Centre on Meat, Hyderabad, Telangana, India; University of Illinois, UNITED STATES

## Abstract

This study describes the muscle transcriptome profile of Bandur breed, a consumer favoured, meat type sheep of India. The transcriptome was compared to the less desirable, unregistered local sheep population, in order to understand the molecular factors related to muscle traits in Indian sheep breeds. Bandur sheep have tender muscles and higher backfat thickness than local sheep. The *longissimus thoracis* transcriptome profiles of Bandur and local sheep were obtained using RNA sequencing (RNA Seq). The animals were male, non-castrated, with uniform age and reared under similar environment, as well as management conditions. We could identify 568 significantly up-regulated and 538 significantly down-regulated genes in Bandur sheep (p≤0.05). Among these, 181 up-regulated and 142 down-regulated genes in Bandur sheep, with a fold change ≥1.5, were considered for further analysis. Significant Gene Ontology terms for the up-regulated dataset in Bandur sheep included transporter activity, substrate specific transmembrane, lipid and fatty acid binding. The down-regulated activities in Bandur sheep were mainly related to RNA degradation, regulation of *ERK1* and *ERK2* cascades and innate immune response. The MAPK signaling pathway, Adipocytokine signaling pathway and PPAR signaling pathway were enriched for Bandur sheep. The highly connected genes identified by network analysis were *CNOT2*, *CNOT6*, *HSPB1*, *HSPA6*, *MAP3K14* and *PPARD*, which may be important regulators of energy metabolism, cellular stress and fatty acid metabolism in the skeletal muscles. These key genes affect the CCR4-NOT complex, PPAR and MAPK signaling pathways. The highly connected genes identified in this study, form interesting candidates for further research on muscle traits in Bandur sheep.

## Introduction

India possesses 6% of the world’s sheep population [1], with 42 registered breeds & several lesser known ones [2]. The economic potential of this ovine biodiversity remains underutilized due to lack of knowledge of their genetic characteristics. Sheep contribute to 7.6% of the total meat production in India [1]. Bandur is a famous mutton type sheep breed of India which is preferred by consumers for its palatability. It fetches a higher price than mutton from other breeds in the same area [3]. It is a registered breed, also known as Mandya or Bannur, mainly distributed in Mandya district of Karnataka. The Bandur animals have a compact body, white coat and a typical reversed U-shaped conformation from the rear [4]. Another population of sheep found in the same area, which is not registered is referred as the local sheep.The local sheep are medium built, heavier than Bandur, with a light brown coat colour. The geographical and management conditions as well as available feed and fodder are similar for both populations. Mutton from Bandur sheep is favoured over local sheep by consumers. The specific organoleptic quality of Bandur meat are attributed to the intramuscular fat content, climate and feed, however, such claims have not been substantiated with scientific studies. The Bandur breed is used for genetic improvement of local sheep population [3]. Despite the local popularity and market potential, no scientific information is available on the uniqueness of its meat quality or muscle traits. Some information is available on the carcass traits for Bandur sheep [5,6,7], but genetic analysis is still lacking.

Since RNA sequencing provides comprehensive data for gene expression studies, it has been widely used to compare transcriptomes across different tissues. RNA sequencing has led to the discovery of differentially expressed (DE) genes for muscle growth, development as well as meat quality of various species including cattle [8], pig [9], goat [10] and sheep [11]. The present study is therefore, an attempt to get an overview of the skeletal muscle transcriptome of Bandur and local sheep. The aim of the study was to compare the gene expression differences in *longissimus thoracis* muscles of Bandur and local sheep. Our findings will provide an insight into the molecular factors related to muscle traits in Indian sheep breeds.

## Materials and methods

### Ethical statement

The samples were collected from animals that had been selected for slaughter for commercial purpose, with prior consultation from slaughter house. The muscle samples from sheep were purchased from local butchers. All ethical norms and guidelines were followed, with approval from Institutional Animal Ethics Committee, ICAR-National Bureau of Animal Genetic Resources, Karnal, Haryana, India (F.No. NBAGR/IAEC/2017, dated 21.01.2017).

### Samples

Four rams of Bandur and four local sheep were identified and selected for analysis. None of the rams were castrated. The selected animals (Bandur and local) were reared under same management conditions. The animals were grazed on uncultivated land and no specific feed was provided to them. All the selected animals were in the two-tooth stage (12–19 months). Body biometry and weight of all the animals were recorded before slaughter. The animals were slaughtered according to standard commercial ‘*halal*’ procedures with 12 hours fasting period before slaughter. All the animals were slaughtered on the same day. Immediately after slaughter, about 600–700 gm of skeletal muscle sample was collected for meat quality analysis. Approximately 5–10 gm of *longissimus thoracis* was immediately stored in RNA*later* (Sigma-Aldrich) for further use.

### Carcass and meat quality analysis

Carcass measurements like hot carcass weight, back fat thickness, fore saddle, hind saddle, foreleg, hind leg, rib eye area, pH, temperature of carcass, water holding capacity (WHC) [12], etc were recorded. Sensory evaluation of fresh and cooked meat was done separately by following 9 point hedonic scale for sensory attributes viz., appearance, flavour, juiciness, texture, mouth coating and overall acceptability [13]. Six semi trained panelists were involved in sensory evaluation of fresh meat. The samples were cooked with 10 per cent water, 1.5 per cent salt (NaCl) and 0.1 per cent turmeric powder in a pressure cooker at 15 lbs psi for 10 minutes. *Longissimus thoracis* muscle was used for physico-chemical analysis. Tenderness of muscles was measured by taking average of shear force for a sample in triplicate according to De Huidobro [14]. Statistical analyses were performed using the SAS software [15]. A *t-test* for independent samples was employed to compare the means. Differences between the means at the 95% (P<0.05) confidence level were considered statistically significant.

### Amino acid and fatty acid analysis

For amino acid analysis the sample was acid hydrolyzed followed by derivatization [16] and analysis in HPLC DAD (Agilent Technologies, Model: 1200 Series). The fat was extracted from the sample, esterified with trans-methylene mixture and methyl esters were separated by liquid-liquid partitioning with petroleum ether and distilled water [17]. Collected organic layer was rotary evaporated and reconstituted in Petroleum ether and injected in GC_FID (Thermo Scientific, Model: Trace GC Ultra), for fatty acid profiling.

### RNA isolation and sequencing

Total RNA was extracted using Trizol method and purified using RNeasy kit (Qiagen). Four biological replicates from Bandur as well as local sheep, with RIN value ranging from 7.0–8.3 (Agilent Bioanalyzer) were used for library preparation by TruSeq RNA Library Prep Kit v2 (Illumina). 100 bp paired end sequencing of the 8 samples was performed on Illumina HiSeq-2000 Platform.

### Data analysis

Quality of the samples was assessed using FastQC (v 0.11.5) [18]. Trimming or filtering on raw reads was done using FastxToolkit (*http*:*//hannonlab*.*cshl*.*edu/fastx_toolkit/index*.*html*), according to the results of FastQC. The reads were mapped against the ovine genome assembly v4.0 (Oar_v4.0),available in NCBI (*https*:*//www*.*ncbi*.*nlm*.*nih*.*gov/assembly/GCF_000298735*.*2*), using TopHat v2.1.1 [19]. The abundance of the transcripts was estimated using Cufflinks v 2.2.1 [20]. All transcripts were assembled using the Cuffmerge and final transcriptome assembly was received as an output. For differential expression estimation Cuffdiff was used. The differential expression results obtained from differential expression estimation were visualized using the R language CummeRbund package [21] and expression plots were placed. The FPKM (Fragments per Kilobase of transcript per Million mapped reads) values were used for quantification of gene expression. The functional annotation and enrichment in pathways of the DE genes was carried out using DAVID [22, 23]. Genemania [24] was used to construct the co-expression network. The network weights reflected the relevance of each gene in the input list. The interaction network was constructed using Consensus Pathway Data Base [25,26] and visualized using Cytoscape ver 3.6.1 [27] along with cytoHubba app [28].

### Validation by quantitative real time PCR (qRT-PCR)

The cDNA was synthesized with 2μg of purified total RNA, using SuperScript III Reverse Transcriptase (Thermo Fisher Scientific), as per manufacturer’s protocol. Primer pairs for five randomly selected DE genes were designed using Primer 3 software [29] or taken from published sequences (S1 Table). Standard PCRs on cDNA were carried out to verify amplicon sizes. The qRT-PCR reaction was performed in triplicate in a final volume of 10μl containing 2μl of cDNA, 8μl of qRT-PCR master mix (5μl of SYBR Green Real-Time master mix, 0.3μl (0.3μM) of each primer, 2.4μl of DNA/RNA-free water) on Roche Light cycler 480 system. A stock solution of 100 μM was prepared for all primers. Each primer was diluted to a concentration of 1 μM/μl, of this, 0.3 μl was used for each reaction. PCR efficiency was estimated by standard curve calculation using four points of a 5-fold dilution series of cDNA. R2 (Pearson Correlation Coefficient) was used to determine the linearity of the curve. An R2 value >0.985 implied consistent efficiency of the reaction. The mean cycle threshold (Ct) values of the genes were normalized to geometric mean of *B2M* and *GAPDH* which were used as reference genes [30]. The data was analyzed by the 2-ΔΔCT method [31].

## Results

### Preliminary analysis of body biometry and phenotypic traits of muscle

The body biometry and some carcass traits of the animals that were used for transcriptome analysis by RNA sequencing were recorded. Details of the body measurements are given in S2 Table. The carcass and meat quality traits of Bandur and local sheep have been summarized in Table 1. Instrumental colour studies indicated that Bandur sheep meat is lighter in colour compared to that of local sheep. The back fat thickness was observed to be significantly greater in Bandur animals as compared to local sheep (P<0.05). The muscles of the Bandur sheep had lower shear force values (16.55N) than local sheep (21.45N). Sensory evaluation of the meat revealed slightly higher juiciness and flavour in Bandur sheep meat but the difference between the two groups was not significant (S3 Table). The fatty acid and amino acid profile revealed that Bandur sheep had a significantly higher level of oleic acid and histidine (p≤0.05) respectively (S4 and S5 Table).

**Table 1:** Comparison of carcass and meat quality traits of Bandur and local sheep of Karnataka.

Variable		Mean values		P values
		Bandur sheep	Local sheep	
**Hot carcass weight (kg)**		12.0 (1.31)	13. 5(1.0)	0.13
**Back fat thickness (cm)**		0.45(0.06)	0.25(0.028)	0.01*
**pH**		5.7(0.035)	5.55(0.09)	0.09
**Temp. of carcass (°C)**		39.75(0.31)	39.45(0.32)	0.26
**WHC (%)**		60.15(1.0)	50.66(1.2)	0.06
**Colour**	L* (lightness)	30.04(1.5)	23.05(1.9)	0.01*
	a* (redness)	10.11(1.0)	13.14(1.4)	0.06
	b*(yellowness)	17.75(0.85)	15.4(0.9)	0.05
**Average tenderness values for muscles (Newton)**		16.55(1.5)	21.45(1.5)	0.04*

(SE in brackets)

*P<0.05

### Summary of RNA seq data

The total number of reads for each library of Bandur (4) and local (4) sheep, ranged from 24,280,035 to 30,330,120 with GC content of 44–50%. Mapping rate with Oar v4.0 ranged from 79–85% (Table 2). The raw sequence data have been submitted to the NCBI Short Read Archive with Accession numbers SRR6260350-SRR6260357. Gene expression levels were evaluated by counting the number of FPKM. For Bandur sheep 6.67% of genes were expressed at >1000 FPKM, 5.81% between 100–1000 FPKM and 87.57% <100 FPKM. For local sheep 2.99% of genes were expressed at >1000 FPKM, 4.97% between 100–1000 FPKM and 92.03% <100 FPKM. A total of 28790 transcripts were observed to be differentially expressed across Bandur and local sheep. Among these, 17168 genes were annotated, of which 8174 were down-regulated and 8994 genes were up-regulated in Bandur sheep. Unique transcripts expressed in local and Bandur sheep were 758 and 1219 respectively.

**Table 2:** Statistics of read mapping to Reference Assembly Oar v4.0.

Properties	Left Reads Input	Left Reads Mapped	Right Reads Input	Right Reads Mapped	Overall Read Mapping Rate
**Local1**	25280035	19743707	25280035	20249308	79.10%
**Local2**	24280035	19958189	24280035	20443789	83.20%
**Local3**	25330120	21074660	25330120	21581262	84.20%
**Local4**	26079105	22062923	26079105	22584505	85.60%
**Bandur1**	27460100	22544742	27460100	23093944	83.10%
**Bandur2**	30330120	24355086	30330120	24961689	81.30%
**Bandur3**	25852630	20966483	25852630	21483536	82.10%
**Bandur4**	27073645	22823083	27073645	23364556	85.30%

### Functional analysis of up-regulated DE genes in Bandur sheep

**Fig 1:**
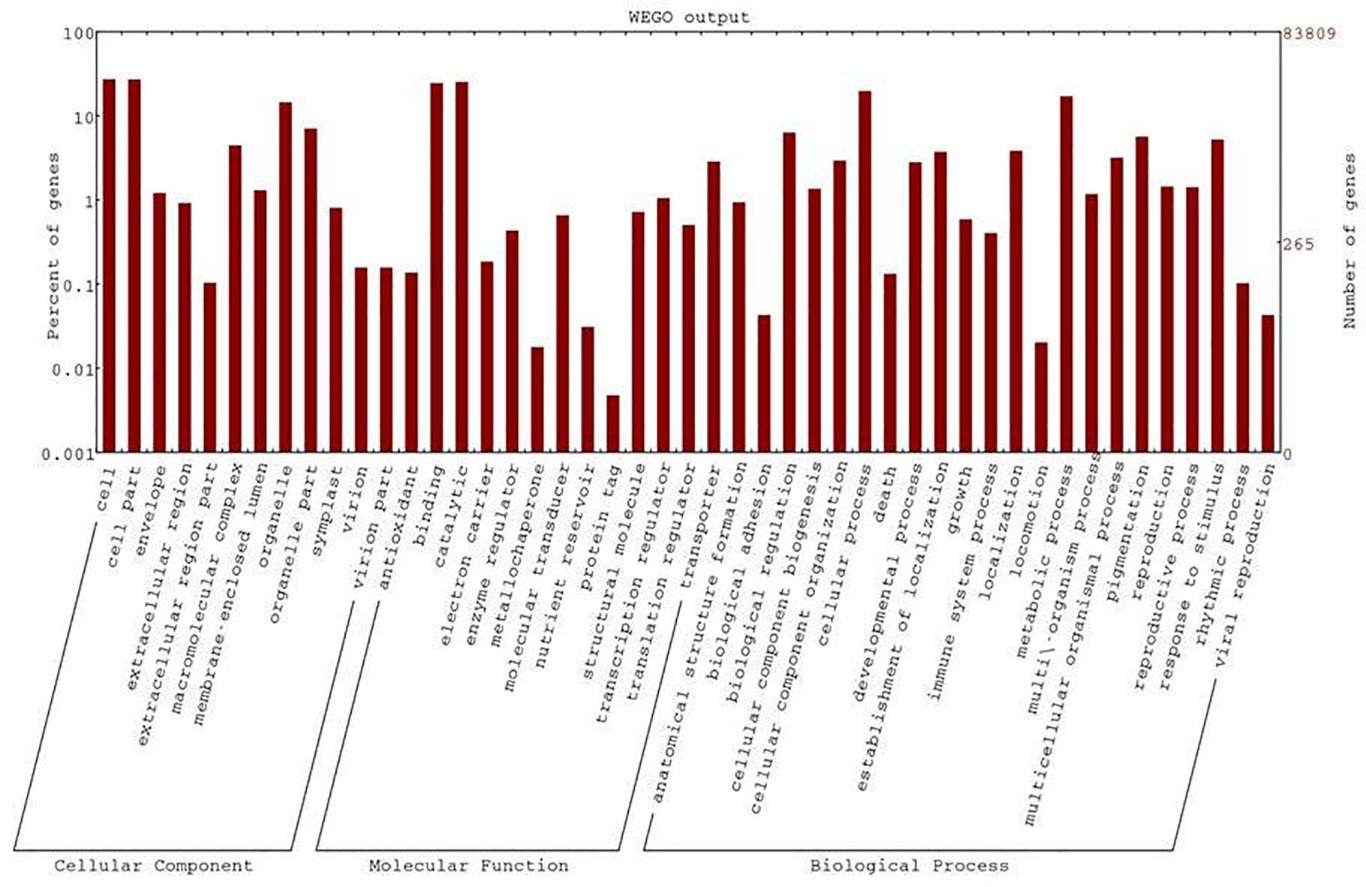
Functional classification of DE genes in Bandur and local sheep.

**Fig 2:**
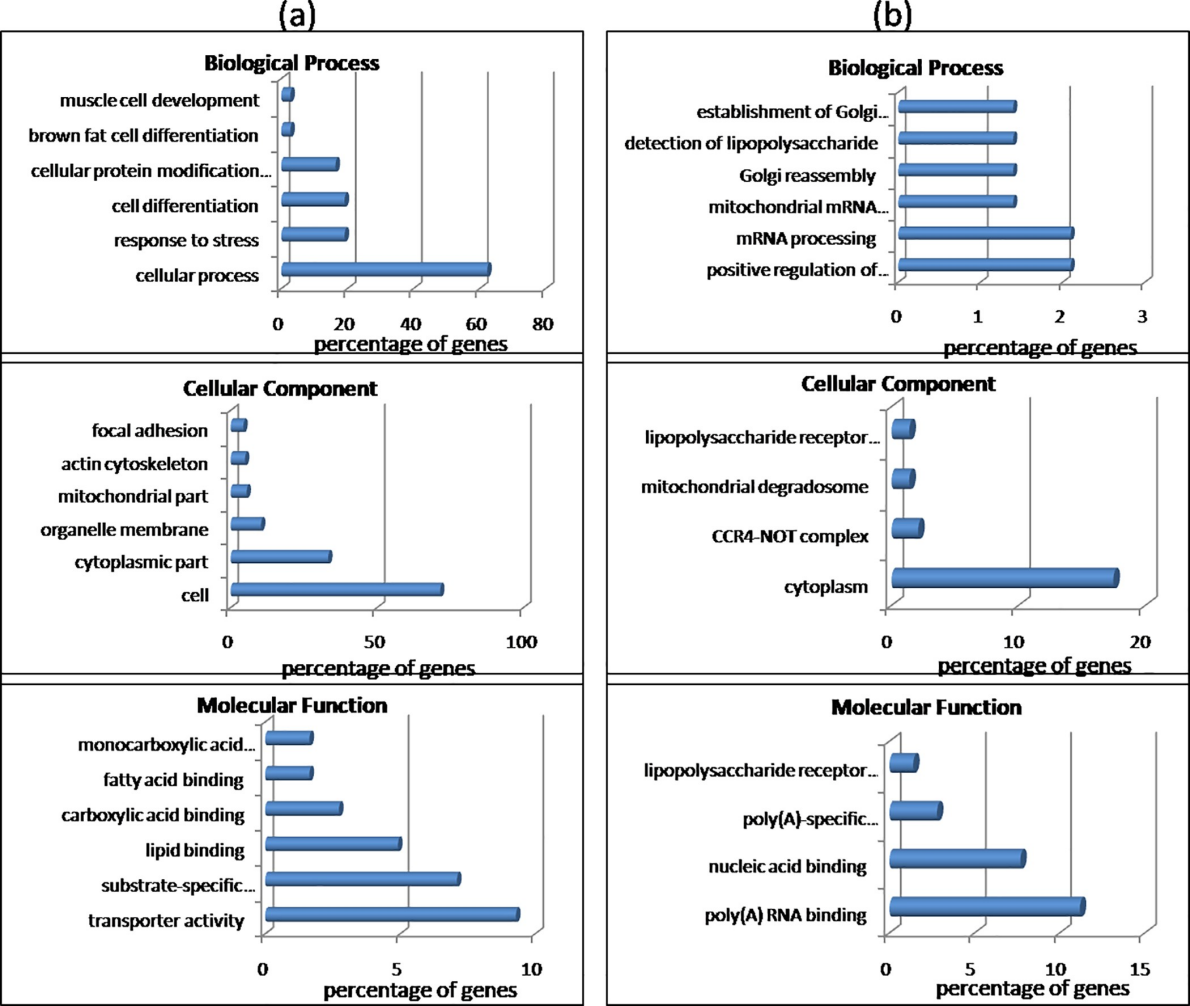
Gene Ontology terms for different categories for (a) up-regulated and (b) down-regulated DE genes in Bandur sheep.

The functional analysis was done to relate the DE genes to cellular components, biological processes and molecular functions. Fig 1 shows the distribution of the identified genes into the three categories. Only 181 up-regulated and 142 down-regulated genes in Bandur sheep (p≤0.05), with a fold change (FC) of ≥1.5 were considered for further analysis. The significant Gene Ontology (GO) terms for the up-regulated dataset, derived using DAVID [22,23], included 56 terms for biological process, 23 terms for cellular components and 9 terms for molecular functions (S6 Table). The significant GO terms for the three categories were further ranked according to percentage of genes in that group. Under biological process GO terms with highest percentage of genes corresponded to cellular process, followed by response to stress, cell differentiation, brown fat cell differentiation and cellular protein modification. The most relevant terms for cellular component were cell, cytoplasmic part, organelle membrane, mitochondrial part, actin cytoskeleton and focal adhesion. Significant GO terms for molecular function for up-regulated dataset included transporter activity, substrate specific transmembrane, lipid and fatty acid binding, among others (Fig 2A). Transporter activity was represented by the genes *ATP2B2*, *CACNG1*, *CHRNA3*, *FABP3*, *FABP4*, *KCNA7*, *OSBP*, *RYR3*, *SCN3B*, *SLC2A1*, *SLC2A4*, *SLC5A3*, *SLC16A3*, *SLC16A7*, *SLC25A13*, *SLC25A33* and *TMCO3*. Genes associated with fat or lipid metabolism in the up-regulated category were *ADIPOQ*, *ADIPOQR2*, *FABP3*, *FABP4*, *AACS*, *ACSM1*, *ACOT11*, *CIDEC*, *FNDC5*, *PPARD*, *TYSND1* and *UNC119*. Genes that exhibited a fold change of ≥ +3.0 included *BCKDK*, *HYAL2*, *TFPT*, *CNEP1R1*, *TNFRS12A*, *BTG2*, *RYR3* and *HSPA6*.

### Functional analysis of down-regulated DE genes in Bandur sheep

The significant GO terms for the 142 down-regulated genes with ≥1.5 FC corresponded to positive regulation of cytoplasmic mRNA processing body assembly, mRNA processing, mitochondrial mRNA catabolic process, Golgi reassembly and detection of lipopolysaccharide for biological process (Fig 2B). Terms like cytoplasm, CCR4-NOT complex, mitochondrial degradosome and lipopolysaccharide receptor complex were relevant in the cellular component category, while poly(A) RNA binding, nucleic acid binding, poly(A)-specific ribonuclease activity and lipopolysaccharide receptor activity were observed to be significant as molecular functions (S7 Table). The down-regulated genes with a fold change of ≥ -3.0 were *VTI1B*, *NUPZ10L*, *DDX39B*, *CDH26*, *ANGPT1*, *CHI3L1* and *HES1*. The down-regulated activities in Bandur sheep were mainly observed to be related to RNA degradation, regulation of *ERK1* and *ERK2* cascades and innate immune response.

### Pathway analysis

The gene clusters identified were further analyzed for their contribution to specific metabolic pathways. A total of 7 annotation clusters were identified using DAVID [22,23], for up-regulated genes, with an enrichment score of >0.5 and p<0.05. The enriched clusters included MAPK signaling pathway, adipocytokine signaling pathway, PPAR signaling pathway and Epstein Barr virus infection. Other prominent pathways included Kelch repeat, Ankyrin repeat and ATP binding. Genes corresponding to adipocytokine signaling pathway included *SLC2A4*, *SLC2A1*, *ADIPOR2* and *ADIPOQ*, while *PPARD*, *FABP3*, *FABP4* and *ADIPOQ* are involved in the PPAR signaling pathway. *HSPA6*, *RRAS*, *HSPB1*, *HSPA1A*, *FLNC*, *MAP3K14*, *CACNG1* and *CD14* genes grouped into the MAPK signaling pathway (S8 Table).

All the down-regulated genes formed 9 enriched clusters with a score of >0.05. The major enriched clusters were RNA degradation, RNA transport and PI3K-Akt signaling pathway. Besides these, ribonuclease activity, innate immune response, CCR4-NOT complex and leucine rich repeat were also identified. Target genes for RNA transport were *PARN*, *CNOT6L*, *PNPT1*, *CNOT2* and *CNOT6*. Genes representing the PI3K-Akt signaling pathway were *YWHAZ*, *EIF4E*, *COL6A6*, *TLR4* and *ANGPT1*. The genes related to ribonuclease activity and nucleotide binding included *PARN*, *CNOT6L*, *CNOT2*, *CNOT6*, *RBM41*, *NT5C3A*, *TIA1*, *SF3B6* and *HNRNPLL*. The genes *CNOT6L*, *CNOT2* and *CNOT6* were associated with CCR4-NOT complex, while *DDX58*, *TLR4* and *MX1* were linked with innate immune response (S9 Table).

### Interaction between DE genes

**Fig 3:**
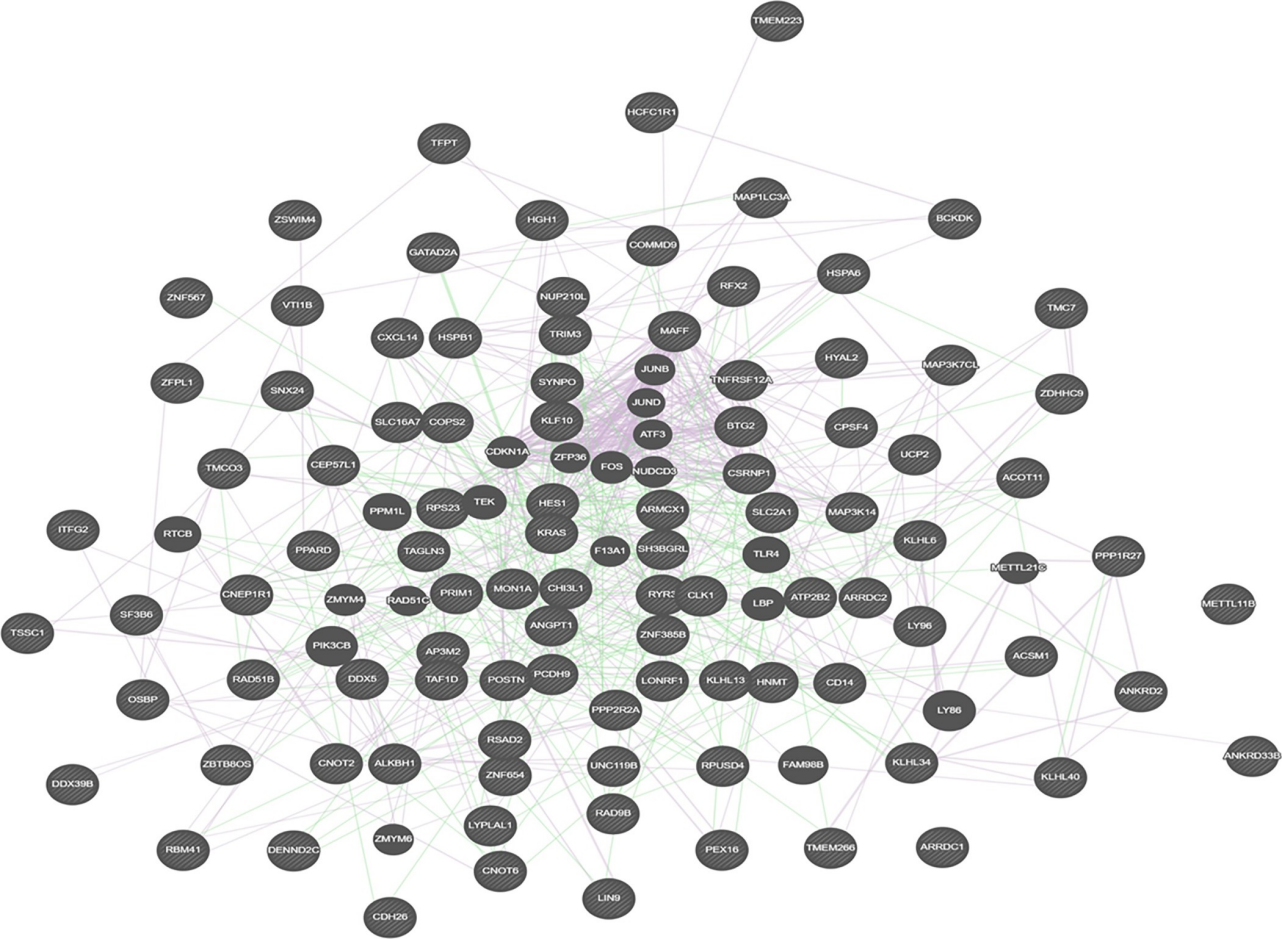
The co-expression network of 99 DE genes based on GeneMANIA.

**Fig 4:**
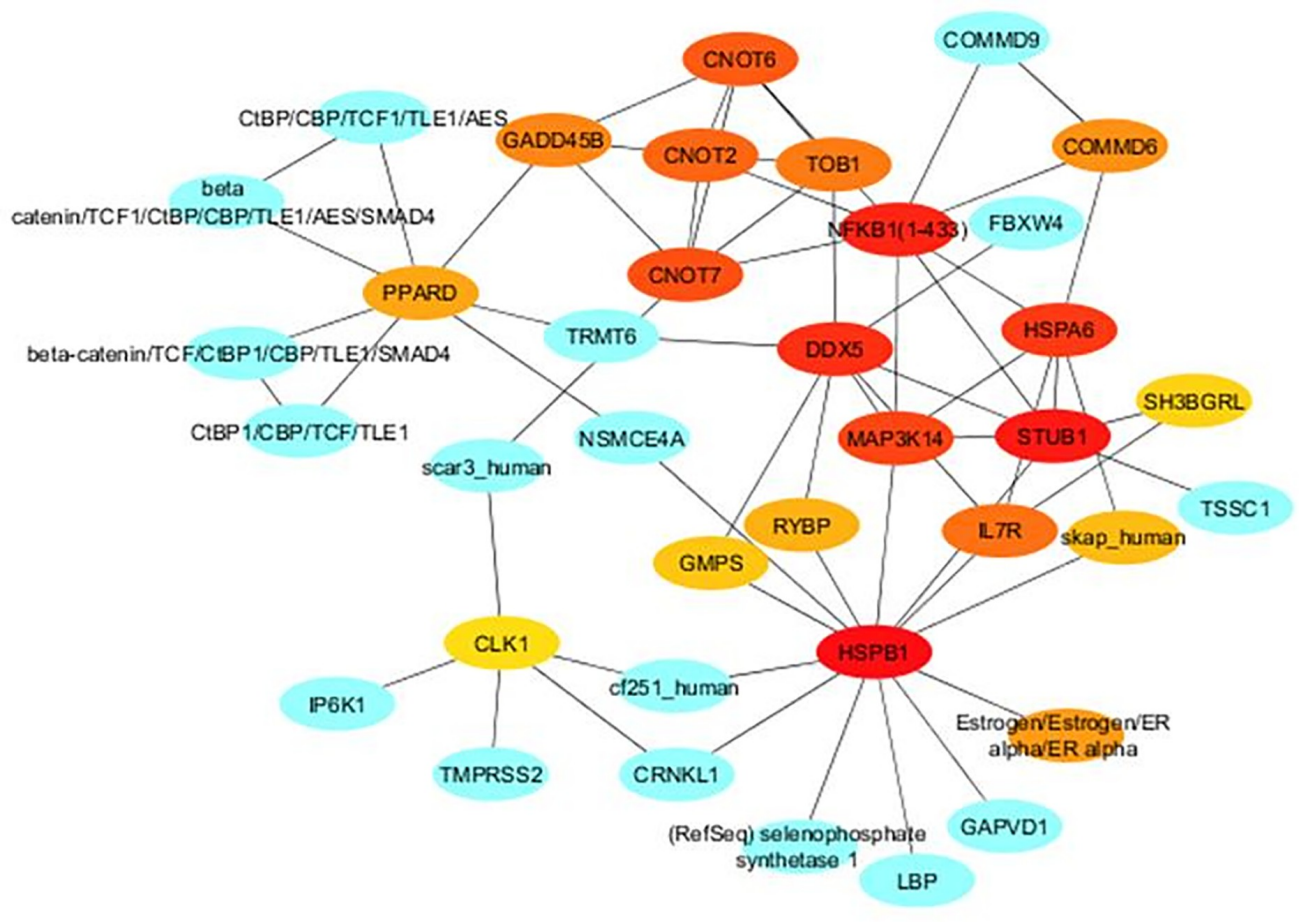
Subnetwork of core DE genes with ≥5.0 degree and a fold change of ≥2.0 (50 nodes and 97 edges). Colour intensity of top 20 genes decreases with increasing order of rank (from light orange to red).

A co-expression network was constructed between 99 DE genes, that were selected based on a threshold of FC ≥ ±2.0 and p<0.05 (Fig 3). A total of 602 interactions were observed. The most relevant genes based on topmost network weights included *HSPB1* (cellular stress), *CNOT2*, *CNOT6* (regulation of gene expression), *KLH13* (muscle cell development), *MAP3K14* (NF-κβ signaling) and *DDX5* (mRNA splicing). Another network was constructed to ascertain the biochemical, protein-protein and gene regulatory interactions between co-expressed genes with ≥5.0 degrees (Fig 4). Among the topmost ranked genes were *MAP3K14*, *CLK1*, *DDX5*, *HSPA6*, *HSPB1*, *CNOT2*, *CNOT4*, *PPARD* (regulates the peroxisomal beta-oxidation pathway of fatty acids) and *SH3BGRL* (muscle development).

### Validation of RNAseq data by qRT-PCR

**Fig 5:**
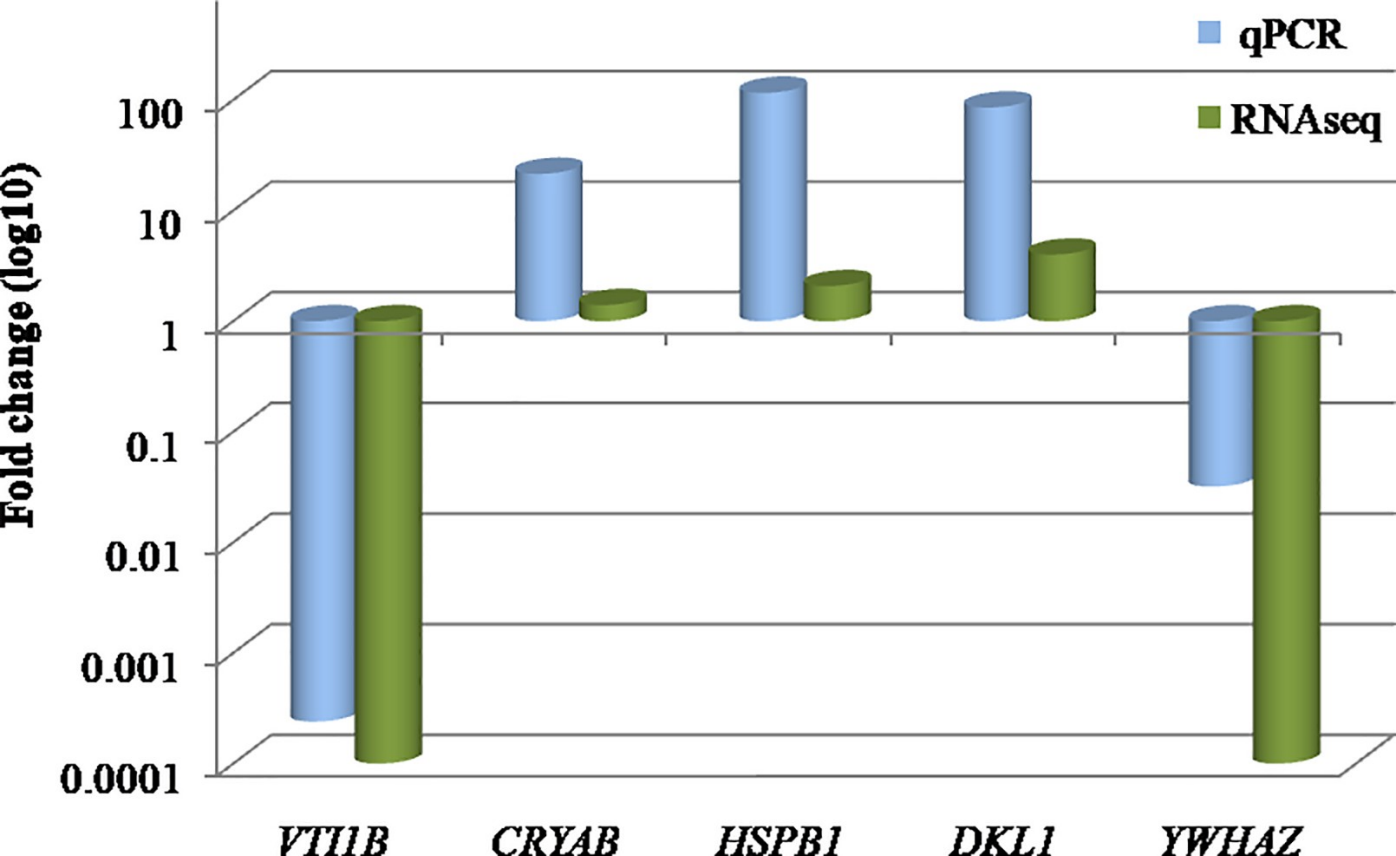
Comparison of fold-change (log_10_) between RNAseq and qRT-PCR data, for selected genes across Bandur and local sheep. qRT-PCR data was normalized by *GAPDH* and *B2M* genes.

Five DE genes namely *HSPB1*, *VTI1B*, *CRYAB*, *DLK1* and *YWHAZ* wereselected at random and their differential expression was validated by qRT-PCR. The results were in concordance with the RNAseq data. The fold change (log_10_) of these genes obtained by qRT-PCR was in agreement to the RNAseq data, although the magnitude was different (Fig 5).

## Discussion

The present study investigated the gene differences in skeletal muscles of phenotypically diverse sheep populations. The animals compared in the study were of similar age, sex and reared under similar environment as well as management conditions. Our results revealed differences in the physico-chemical traits of meat from both local and Bandur sheep. A total of 99 highly significant DE genes with fold change ≥ ±2.0 and p<0.05, were identified in our study. Most of the genes identified in our study were related to muscle development or differentiation, fat metabolism and to a lesser extent to energy metabolism, cellular stress and immune response. Molecular events that occur during muscle development, fat deposition, post-mortem proteolysis and energy metabolism are important for underpinning genes underlying meat quality. Therefore, in this study we focused the analysis on genes and pathways that are known to be associated with muscle development, lipid metabolism, tenderness of muscles and postmortem proteolysis.

### Genes related to muscle development

The skeletal muscle transcriptome analysis of Indian sheep revealed several genes that may contribute to muscle development. Three members of the Kelch superfamily (*KLHL6*, *KLHL34*, *KLHL40*) were observed to be up-regulated (>2.0 fold) in Bandur sheep. Recent reports have underlined the role of Kelch proteins in muscle cell development as well as disease [32]. Apart from the Kelch family, studies have also implicated *ANKRD2* and the MAPK pathway in myogenesis [33,34]. The MAPK signaling pathway was observed to be up-regulated while RNA degradation pathway was down-regulated. A transcriptional activator *MYOG* known to regulate muscle differentiation and atrophy [35] was also over expressed in Bandur sheep.

### Genes related to intramuscular fat (IMF)

Consumer preference for Bandur meat is due to its flavor. IMF contributes to meat quality and consumer acceptance. Fat plays a major role in the palatability of meat [36]. Differentially expressed genes associated with fatty acid and lipid metabolism have been delineated in beef [37]. In this study, several DE genes associated with fatty acid metabolism were over-expressed in Bandur sheep. Among these the *FABP3*, *FABP4* and *ADIPOQ* genes play an important role in the regulation of lipid and glucose homeostasis in adipocytes [38, 39]. The *FABP4* gene falls into a significant QTL interval for beef marbling on bovine chromosome 14 [40]. An SNP in the *FABP4* gene was reported to be associated with meat tenderness in Chinese sheep breeds [41]. Recently, polymorphisms in *ADIPOQ* gene have been associated with growth and carcass traits in sheep [42]. Genes that are indirectly associated with synthesis and degradation of fatty acids (*ACSS1* and *BDH*) [37] were observed to be up-regulated in Bandur sheep.

### Genes related to tenderness

Tenderness is determined by the muscle fibres, intramuscular fat content and post mortem processing [43]. Enriched GOs in Bandur included muscle development and differentiation, lipid metabolism and regulation as well as ion binding and response to stress. Genes involved in cell cycle, energy metabolism and muscle development have been associated with tenderness in pigs [44]. Tenderness in muscles of cattle has also been associated to several genes belonging to the heat shock protein (*HSP*) family, voltage gated ion channels, fatty acid and energy metabolism [8,45]. Proteomic studies have also underlined the significance of small Hsps in tenderness of muscle type and breeds [46]. The expression of *HSPB1* was negatively correlated with beef tenderness [45]. Expression of *HSPB1* gene has also been associated with intramuscular fat content in cattle [47]. Some of these genes like *HSPB1*, *DNAJB5*, *HSPA6* were over expressed in Bandur sheep consistent with other studies on beef [48,49]. Although several studies have investigated the role of small heat shock proteins in meat tenderness, the mechanism of their regulation is still not well understood [50].

### Genes related to postmortem proteolysis

Meat tenderness is a complex trait which involves the interplay of muscle characteristics as well post mortem ageing of meat. The expression of these genes has mostly been investigated in cattle but their involvement in sheep muscles is less explored. Cellular stress and ion channels influence the post mortem mechanisms affecting muscle phenotype [51]. Four genes controlling these ion channels were observed to be up-regulated in Bandur sheep. These include *CACNG1* (calcium voltage gated channel auxiliary subunit gamma1), *KCNMA1* (potassium calcium activated channel subfamily M), *RYR3* (ryanodine receptor family) and *SCN3B* (sodium voltage gated channel beta subunit 3). A mutation in the *RYR1* gene results in pale, soft, and exudative (PSE) meat in pigs, which is undesirable [52]. Not much information is available about *RYR3* gene although it is believed to be involved in release of intracellular calcium ions [53].

It is intriguing that some genes that have not been reported earlier were over-expressed in Bandur sheep. These are mainly involved in calcium ion channels, myogenesis and lipid metabolism. Notable among them are *RYR3* which belongs to the family of ryanodine receptors which modulates release of calcium ions from intracellular storage for use in many cellular processes [54,55]. *HES1* modulates myoblast differentiation [56], *CNEP1R1* [57], *UCP2* [58] and *ACOT11* [59] are involved in lipid metabolism.

### Highly connected DE genes

Differential expression alone does not provide information of the functional interactions of a gene. To understand the relation between the DE genes and their regulatory factors, it is important to analyze their connectivity to other molecules or regulators [60]. The network analysis identified highly connected genes like *CNOT2*, *CNOT6*, *HSPB1*, *HSPA6*, *KLH13*, *MAP3K14* and *PPARD*. These may be important regulators of energy metabolism, cellular stress and fatty acid metabolism in skeletal muscles. These genes would be expected to have maximum impact on the relevant pathways. Except for *CNOT2* and *CNOT6*, the other key genes were up-regulated in Bandur sheep. Recent studies have elucidated the role of the CCR4-NOT complex (*CNOT2*, *CNOT6*) in regulation of RNA expression and lipogenesis as well as its deficiency in regulating apoptosis [61]. The up-regulated key genes are constituents of the PPAR and MAPK signaling pathways. Although it is too early to pinpoint the interplay of these genes or pathways in *longissimus thoracis* muscles of sheep, functional homology to other livestock species, implicates them in myogenesis, lipid metabolism and cellular stress.

### Conclusions

The study reports the muscle transcriptome profile of Bandur sheep in comparison to local sheep of similar age, sex and management conditions. Several DE genes related to energy metabolism, lipid metabolism, muscle development, cellular stress and voltage gated ion transport were identified in this study. The highly connected DE genes identified in our study, form interesting candidates for further research on muscle characteristics in Indian sheep.

## Supporting information

S1 Table Details of primers used for quantitative PCR. (DOCX)

S2 Table Average body measurements and carcass traits of Bandur and local sheep. (DOCX)

S3 Table Sensory evaluation of fresh meat and cooked meat by following 9 point hedonic scale. (DOCX)

S4 Table Fatty acid profile of Bandur and local sheep on 100% fat basis. (DOCX)

S5 Table Amino acid profile of Bandur and local sheep. (DOCX)

S6 Table Gene ontology terms identified for biological process, cellular components and molecular functions for up-regulated genes in Bandur sheep. (DOCX)

S7 Table Gene ontology terms identified for biological process, cellular components and molecular functions for down-regulated genes in Bandur sheep. (DOCX)

S8 Table Pathway terms for up-regulated genes in Bandur sheep. (DOCX)

S9 Table Pathway terms for down-regulated genes in Bandur sheep. (DOCX)
