# Supplementary material for: Transcriptome profiling of longissimus thoracis muscles identifies highly connected differentially expressed genes in meat type sheep of India
Source: PLoS One. 2019 Jun 6;14(6):e0217461. doi: 10.1371/journal.pone.0217461 (PMC6553717; doi:10.1371/journal.pone.0217461)
Supplement: S1 Table — (DOCX) [file pone.0217461.s001.docx]

| **SN** | **Gene** | **Primer** | **Sequence** | **Melting Temp.(°C)** | **Amplicon size (bp)** | **Reference** |
| --- | --- | --- | --- | --- | --- | --- |
| 1. | ***GAPDH*** | F: | ggtcggagtgaacggatttg | 61 | 83 | 62 |
|  |  | R: | tggcaacgatgtccactttg | 61 |  |  |
| 2. | ***B2M*** | F: | ggcctgctgtcgctgtct | 60 | 78 | 62 |
|  |  | R: | ttctggcgggtgtcttgagt | 60 |  |  |
| 3. | ***VTI1B*** | F | gcctttgacagccactc | 60 | 150 | This study |
|  |  | R | cgctcaatactctgggtg | 60 |  |  |
| 4. | ***CRYAB*** | F | cgccattacttcatccctgt | 60 | 189 | 63 |
|  |  | R | tcactggtggggaacttttc | 60 |  |  |
| 5. | ***HSPB1*** | F | cgttgcttcactcgcaaata | 60 | 210 | 63 |
|  |  | R | tacttgtttccggctgttcg | 60 |  |  |
| 6. | **YWHAZ** | F: | tgtaggagcccgtaggtcatct | 62 | 102 | 64 |
|  |  | R: | ttctctctgtattctcgagccatct | 62 |  |  |
| 7. | ***DLK1*** | F: | cgtcttcctcaacaagtgcga | 60 | 102 | 65 |
|  |  | R: | tcctccccgctgttgtagtg | 60 |  |  |

**S1 Table. Details of primers used for quantitative PCR**

62. French MC, LittlejohnRP,Greer GJ, Bain WE, McEwan JC, TisdallDJ*.* Growth hormone and ghrelin receptor genes are differentially expressed between genetically lean and fat selection lines of sheep. J Anim Sci.2006;84:324-331.

63. Bernard C, Cassar-Malek I, Le Cunff M, Dubroeucq H, Renand G, Hocquette JF.  New indicators of beef sensory quality revealed by expression of specific genes. J Agric Food Chem. 2007; 55(13):5229-37.

64. Garcia-Crespo D, Juste RA, Hurtado A. Selection of ovine housekeeping genes for normalization by real-time RT-PCR; analysis of PrP gene expression and genetic susceptibility to scrapie. BMC Vet Res. 2005; 1-3.

65. Su R, Sun W, Li D, Wang QZ, Lv XY, Musa HH et al. Association between DLK1 and IGF-I gene expression and meat quality in sheep. Genet Mol Res. 2014; 13**:**10308-19.
