# Supplementary material for: Transcriptome profiling of longissimus thoracis muscles identifies highly connected differentially expressed genes in meat type sheep of India
Source: PLoS One. 2019 Jun 6;14(6):e0217461. doi: 10.1371/journal.pone.0217461 (PMC6553717; doi:10.1371/journal.pone.0217461)
Supplement: S2 Table — (DOCX) [file pone.0217461.s002.docx]

**S2 Table. Average body measurements and carcass traits of Bandur and local sheep**

| **Linear Body Measurements** | **Bandur Sheep (4)** | **SE** | **Local Sheep (4)** | **SE** |
| --- | --- | --- | --- | --- |
| **Pre slaughter weight (kg)** | 22.4 | 1.22 | 27.0 | 1.77 |
| **Wither Height (cm)** | 45.0 | 0.91 | 56.5 | 0.81 |
| **Rump Height (cm)** | 46.8 | 1.31 | 58.5 | 1.65 |
| **Body Length (cm)** | 55.3 | 1.43 | 67.0 | 1.45 |
| **Chest Girth (cm)** | 63.0 | 1.06 | 70.3 | 1.12 |
| **Chest Depth (cm)** | 35.8 | 1.65 | 36.8 | 1.25 |
| **Distance b/w Hip Bones (cm)** | 15.1 | 0.4 | 17.3 | 0.47 |
| **Shoulder Width (cm)** | 44.3 | 1.25 | 45.5 | 1.84 |
| **Fore Limb Length (cm)** | 25.8 | 0.45 | 37.3 | 0.85 |
| **Distance b/w wither to pin bones** | 55.3 | 1.02 | 64.3 | 1.32 |
| **Head Length (cm)** | 18.5 | 0.86 | 20.0 | 0.91 |
| **Ear Length (cm)** | 15.3 | 0.25 | 16.5 | 0.86 |
| **Head (kg)** | 1.45 | 0.11 | 1.72 | 0.12 |
| **Fore leg (kg)** | 0.24 | 0.02 | 0.33 | 0.02 |
| **Hind leg (kg)** | 0.21 | 0.01 | 0.29 | 0.02 |
| **Skin (kg)** | 2.42 | 0.28 | 2.80 | 0.21 |
| **Omental fat (kg)** | 0.20 | 0.05 | 0.28 | 0.07 |
| **Intestine without content (kg)** | 0.76 | 0.07 | 1.07 | 0.11 |
| **Stomach without content (kg)** | 0.66 | 0.05 | 0.89 | 0.06 |
| **Lungs and trachea (kg)** | 0.22 | 0.02 | 0.31 | 0.03 |
| **Liver (kg)** | 0.42 | 0.04 | 0.61 | 0.04 |
| **Spleen (kg)** | 0.09 | 0.01 | 0.14 | 0.01 |
| **Heart (kg)** | 0.12 | 0.01 | 0.17 | 0.02 |
| **Kidney (kg)** | 0.06 | 0.002 | 0.08 | 0.01 |
| **Kidney fat (kg)** | 0.14 | 0.03 | 0.12 | 0.03 |
| **Testes (kg)** | 0.04 | 0.001 | 0.26 | 0.01 |

SE –Standard Error
