# Supplementary material for: Transcriptome profiling of longissimus thoracis muscles identifies highly connected differentially expressed genes in meat type sheep of India
Source: PLoS One. 2019 Jun 6;14(6):e0217461. doi: 10.1371/journal.pone.0217461 (PMC6553717; doi:10.1371/journal.pone.0217461)
Supplement: S3 Table — (DOCX) [file pone.0217461.s003.docx]

**S3 Table. Sensory evaluation of Fresh Meat and Cooked Meat by following 9 point hedonic scale**

Note: Means bearing same superscripts are not significantly different at P<0.05

|  | **Appearance** | | **Juiciness** | | **Texture** | | **Flavour** | | **Mouth Coating** | | **Overall Acceptance** | |
| --- | --- | --- | --- | --- | --- | --- | --- | --- | --- | --- | --- | --- |
|  | **Local** | **Bandur** | **Local** | **Bandur** | **Local** | **Bandur** | **Local** | **Bandur** | **Local** | **Bandur** | **Local** | **Bandur** |
| **Fresh Meat** | 7.28±0.30^a^ | 6.68±0.10^a^ | - | - | 7.13±0.15^a^ | 6.60±0.14^a^ | 6.79±0.12^a^ | 6.74±0.16^a^ | - | - | 7.03±0.16^a^ | 6.69±0.12^a^ |
| **Cooked Meat** | 6.77± 0.19^a^ | 6.73±0.15^a^ | 6.40±0.07^a^ | 6.47±0.09^a^ | 6.47±0.08^a^ | 6.46±0.09^a^ | 6.37±0.18^a^ | 6.47±0.16^a^ | 6.05±0.18^a^ | 6.29±0.13^a^ | 6.48±0.14^a^ | 6.49±0.15^a^ |
