# Supplementary material for: Transcriptome profiling of longissimus thoracis muscles identifies highly connected differentially expressed genes in meat type sheep of India
Source: PLoS One. 2019 Jun 6;14(6):e0217461. doi: 10.1371/journal.pone.0217461 (PMC6553717; doi:10.1371/journal.pone.0217461)
Supplement: S4 Table — (DOCX) [file pone.0217461.s004.docx]

**S4 Table. Fatty Acid Profile of Bandur and Local sheep on 100% Fat basis**

| **Saturated Fatty Acids** | **Bandur** | **Local** | **P** |
| --- | --- | --- | --- |
| Myristic Acid | 0.04 | 0.08 | 0.17 |
| Pentadecanoic Acid | 0.02 | 0.03 | 0.57 |
| Palmitic Acid | 0.27 | 0.28 | 0.58 |
| Stearic Acid | 0.21 | 0.21 | 0.98 |
| Arachidic Acid | 0.00 | 0.00 | 0.16 |
| Average | 0.11 | 0.12 | 0.87 |
| **Mono Unsaturated Fatty Acids** | | |  |
| Myristoleic Acid | 0.02 | 0.03 | 0.80 |
| Palmetoleic Acid | 0.02 | 0.02 | 0.49 |
| Oleic Acid | 0.39 | 0.32 | 0.05 |
| Average | 0.14 | 0.12 | 0.89 |
| **Poly Unsaturated Fatty Acids** | | |  |
| Linoleic Acid | 0.02 | 0.02 | 0.82 |
| Linolenic Acid | 0.01 | 0.01 | 0.15 |
| Cis-5,8,11,14,17-Eicosapentaenoic Acid | 0.00 | 0.00 | 1.00 |
| Cis-4,7,10,13,16,19-docosahexanoic Acid | 0.00 | 0.00 | - |
| Average | 0.01 | 0.01 | 1.00 |
