# Supplementary material for: Transcriptome profiling of longissimus thoracis muscles identifies highly connected differentially expressed genes in meat type sheep of India
Source: PLoS One. 2019 Jun 6;14(6):e0217461. doi: 10.1371/journal.pone.0217461 (PMC6553717; doi:10.1371/journal.pone.0217461)
Supplement: S5 Table — (DOCX) [file pone.0217461.s005.docx]

**S5 Table.** **Amino acid profile of Bandur and Local sheep**

| **(mg/100g)** | **Bandur** | **Local** | **P** |
| --- | --- | --- | --- |
| **Histidine** | 158.47 | 51.94 | 0.01 |
| **Serine** | 184.32 | 207.57 | 0.35 |
| **Arginine** | 372.11 | 356.71 | 0.73 |
| **Glycine** | 530.17 | 505.24 | 0.67 |
| **Aspartic Acid** | 975.56 | 979.13 | 0.98 |
| **Glutamic Acid** | 1205.80 | 1259.77 | 0.72 |
| **Threonine** | 165.96 | 182.00 | 0.51 |
| **Alanine** | 501.21 | 512.60 | 0.87 |
| **Proline** | 326.31 | 340.63 | 0.77 |
| **Cysteine** | 22.24 | 22.46 | 0.95 |
| **Lysine** | 475.45 | 455.56 | 0.83 |
| **Tyrosine** | 187.98 | 225.97 | 0.30 |
| **Methionine** | 141.82 | 159.96 | 0.48 |
| **Valine** | 116.93 | 128.37 | 0.57 |
| **Isoleucinele** | 90.91 | 115.24 | 0.22 |
| **Leucine** | 460.04 | 514.84 | 0.49 |
| **Phenylalanine** | 199.78 | 231.89 | 0.38 |
