# Supplementary material for: Transcriptome profiling of longissimus thoracis muscles identifies highly connected differentially expressed genes in meat type sheep of India
Source: PLoS One. 2019 Jun 6;14(6):e0217461. doi: 10.1371/journal.pone.0217461 (PMC6553717; doi:10.1371/journal.pone.0217461)
Supplement: S6 Table — (DOCX) [file pone.0217461.s006.docx]

**S6 Table. Gene ontology terms identified for biological process, cellular components and molecular functions for up-regulated genes in Bandur sheep**

| **Term** | **Biological Process** |
| --- | --- |
| GO:0050873 | brown fat cell differentiation |
| GO:0032640 | tumor necrosis factor production |
| GO:0032680 | regulation of tumor necrosis factor production |
| GO:1903555 | regulation of tumor necrosis factor superfamily cytokine production |
| GO:0071706 | tumor necrosis factor superfamily cytokine production |
| GO:0032787 | monocarboxylic acid metabolic process |
| GO:0006950 | response to stress |
| GO:0048747 | muscle fiber development |
| GO:0045927 | positive regulation of growth |
| GO:0007519 | skeletal muscle tissue development |
| GO:0019752 | carboxylic acid metabolic process |
| GO:0043436 | oxoacid metabolic process |
| GO:0043407 | negative regulation of MAP kinase activity |
| GO:0060538 | skeletal muscle organ development |
| GO:0055001 | muscle cell development |
| GO:0009628 | response to abiotic stimulus |
| GO:0044281 | small molecule metabolic process |
| GO:0045773 | positive regulation of axon extension |
| GO:0044723 | single-organism carbohydrate metabolic process |
| GO:0035914 | skeletal muscle cell differentiation |
| GO:0006082 | organic acid metabolic process |
| GO:0061061 | muscle structure development |
| GO:0044283 | small molecule biosynthetic process |
| GO:0043412 | macromolecule modification |
| GO:0030225 | macrophage differentiation |
| GO:0044710 | single-organism metabolic process |
| GO:0043409 | negative regulation of MAPK cascade |
| GO:0044262 | cellular carbohydrate metabolic process |
| GO:0045936 | negative regulation of phosphate metabolic process |
| GO:0010563 | negative regulation of phosphorus metabolic process |
| GO:0016310 | phosphorylation |
| GO:0022411 | cellular component disassembly |
| GO:0009987 | cellular process |
| GO:0044763 | single-organism cellular process |
| GO:0006469 | negative regulation of protein kinase activity |
| GO:0016051 | carbohydrate biosynthetic process |
| GO:0001817 | regulation of cytokine production |
| GO:0033554 | cellular response to stress |
| GO:0048639 | positive regulation of developmental growth |
| GO:0016043 | cellular component organization |
| GO:0006464 | cellular protein modification process |
| GO:0036211 | protein modification process |
| GO:0030154 | cell differentiation |
| GO:0006631 | fatty acid metabolic process |
| GO:0071901 | negative regulation of protein serine/threonine kinase activity |
| GO:0005975 | carbohydrate metabolic process |
| GO:0014706 | striated muscle tissue development |
| GO:0097190 | apoptotic signaling pathway |
| GO:0033673 | negative regulation of kinase activity |
| GO:0042692 | muscle cell differentiation |
| GO:0031324 | negative regulation of cellular metabolic process |
| GO:0019220 | regulation of phosphate metabolic process |
| GO:0071840 | cellular component organization or biogenesis |
| GO:0051174 | regulation of phosphorus metabolic process |
| GO:0001816 | cytokine production |
| GO:0055002 | striated muscle cell development |
|  |  |
| **Term** | **Cellular Component** |
| GO:0043226 | organelle |
| GO:0005622 | intracellular |
| GO:0043227 | membrane-bounded organelle |
| GO:0044424 | intracellular part |
| GO:0043229 | intracellular organelle |
| GO:0005623 | cell |
| GO:0044444 | cytoplasmic part |
| GO:0044464 | cell part |
| GO:0015629 | actin cytoskeleton |
| GO:0005829 | cytosol |
| GO:0031967 | organelle envelope |
| GO:0031975 | envelope |
| GO:0043231 | intracellular membrane-bounded organelle |
| GO:0031226 | intrinsic component of plasma membrane |
| GO:0070603 | SWI/SNF superfamily-type complex |
| GO:0031090 | organelle membrane |
| GO:0005925 | focal adhesion |
| GO:0005924 | cell-substrate adherens junction |
| GO:0044429 | mitochondrial part |
| GO:0030055 | cell-substrate junction |
| GO:0005615 | extracellular space |
| GO:0044422 | organelle part |
| GO:0000790 | nuclear chromatin |
|  |  |
| **Term** | **Molecular Function** |
| GO:0016878 | acid-thiol ligase activity |
| GO:0031406 | carboxylic acid binding |
| GO:0005504 | fatty acid binding |
| GO:0016405 | CoA-ligase activity |
| GO:0016877 | ligase activity, forming carbon-sulfur bonds |
| GO:0033293 | monocarboxylic acid binding |
| GO:0008289 | lipid binding |
| GO:0005215 | transporter activity |
| GO:0022891 | substrate-specific transmembrane transporter activity |
