# Supplementary material for: Transcriptome profiling of longissimus thoracis muscles identifies highly connected differentially expressed genes in meat type sheep of India
Source: PLoS One. 2019 Jun 6;14(6):e0217461. doi: 10.1371/journal.pone.0217461 (PMC6553717; doi:10.1371/journal.pone.0217461)
Supplement: S7 Table — (DOCX) [file pone.0217461.s007.docx]

**S7 Table. Gene ontology terms identified for biological process, cellular components and molecular functions for down-regulated genes in Bandur sheep**

| **Term** | **Biological Process** |
| --- | --- |
| GO:0010606 | positive regulation of cytoplasmic mRNA processing body assembly |
| GO:0032755 | positive regulation of interleukin-6 production |
| GO:0000958 | mitochondrial mRNA catabolic process |
| GO:0000962 | positive regulation of mitochondrial RNA catabolic process |
| GO:0072012 | glomerulus vasculature development |
| GO:0061157 | mRNA destabilization |
| GO:0090168 | Golgi reassembly |
| GO:0032497 | detection of lipopolysaccharide |
| GO:0006397 | mRNA processing |
| GO:0000398 | mRNA splicing, via spliceosome |
| GO:0051770 | positive regulation of nitric-oxide synthase biosynthetic process |
| GO:0051683 | establishment of Golgi localization |
| GO:0046784 | viral mRNA export from host cell nucleus |
| GO:0071850 | mitotic cell cycle arrest |
| GO:0032725 | positive regulation of granulocyte macrophage colony-stimulating factor production |
| GO:0045944 | positive regulation of transcription from RNA polymerase II promoter |
| GO:0006470 | protein dephosphorylation |
| GO:0032727 | positive regulation of interferon-alpha production |
| GO:0034142 | toll-like receptor 4 signaling pathway |
|  |  |
| **Term** | **Cellular Component** |
| GO:0030014 | CCR4-NOT complex |
| GO:0045025 | mitochondrial degradosome |
| GO:0005737 | cytoplasm |
| GO:0046696 | lipopolysaccharide receptor complex |
| GO:0005829 | cytosol |
|  |  |
| **Term** | **Molecular Function** |
| GO:0004535 | poly(A)-specific ribonuclease activity |
| GO:0044822 | poly(A) RNA binding |
| GO:0003676 | nucleic acid binding |
| GO:0001875 | lipopolysaccharide receptor activity |
| GO:0000166 | nucleotide binding |
| GO:0004004 | ATP-dependent RNA helicase activity |
