# Supplementary material for: Transcriptome profiling of longissimus thoracis muscles identifies highly connected differentially expressed genes in meat type sheep of India
Source: PLoS One. 2019 Jun 6;14(6):e0217461. doi: 10.1371/journal.pone.0217461 (PMC6553717; doi:10.1371/journal.pone.0217461)
Supplement: S8 Table — (DOCX) [file pone.0217461.s008.docx]

**S8 Table. Pathway terms for up-regulated genes in Bandur sheep**

| **Category** | **Term** | **Count** | **%** | **PValue** | **Genes** |
| --- | --- | --- | --- | --- | --- |
| **KEGG_PATHWAY** | oas04010:MAPK signaling pathway | 10 | 5.464481 | 3.03E-04 | RPS6KA2, MAP2K3, HSPA6, RRAS, HSPB1, HSPA1A, FLNC, MAP3K14, CACNG1, CD14 |
| **KEGG_PATHWAY** | oas05134:Legionellosis | 4 | 2.185792 | 0.019882 | HSF1, HSPA6, HSPA1A, CD14 |
| **KEGG_PATHWAY** | oas05164:Influenza A | 6 | 3.278689 | 0.021835 | MAVS, EIF2AK1, MAP2K3, HSPA6, HSPA1A, CPSF4 |
| **KEGG_PATHWAY** | oas03320:PPAR signaling pathway | 4 | 2.185792 | 0.022309 | PPARD, FABP3, FABP4, ADIPOQ |
| **KEGG_PATHWAY** | oas00650:Butanoate metabolism | 3 | 1.639344 | 0.025652 | ACSM1, AACS, BDH1 |
| **KEGG_PATHWAY** | oas04920:Adipocytokine signaling pathway | 4 | 2.185792 | 0.026698 | SLC2A4, SLC2A1, ADIPOR2, ADIPOQ |
| **KEGG_PATHWAY** | oas05169:Epstein-Barr virus infection | 6 | 3.278689 | 0.029294 | EIF2AK1, MAP2K3, HSPA6, HSPB1, HSPA1A, MAP3K14 |
