# Supplementary material for: Transcriptome profiling of longissimus thoracis muscles identifies highly connected differentially expressed genes in meat type sheep of India
Source: PLoS One. 2019 Jun 6;14(6):e0217461. doi: 10.1371/journal.pone.0217461 (PMC6553717; doi:10.1371/journal.pone.0217461)
Supplement: S9 Table — (DOCX) [file pone.0217461.s009.docx]

**S9 Table. Pathway terms for down-regulated genes in Bandur sheep**

| **Category** | **Term** | **Count** | **%** | **PValue** | **Genes** |
| --- | --- | --- | --- | --- | --- |
| **KEGG_PATHWAY** | oas03018:RNA degradation | 5 | 3.496503 | 0.003459 | PARN, CNOT6L, PNPT1, CNOT2, CNOT6 |
| **KEGG_PATHWAY** | oas05164:Influenza A | 7 | 4.895105 | 0.004136 | DDX58, IL18, DDX39B, RSAD2, TLR4, IL33, MX1 |
| **KEGG_PATHWAY** | oas03013:RNA transport | 6 | 4.195804 | 0.014362 | EIF4E, RAN, EIF3E, DDX39B, THOC7, NUP210L |
| **KEGG_PATHWAY** | oas04151:PI3K-Akt signaling pathway | 8 | 5.594406 | 0.025064 | YWHAZ, EIF4E, KRAS, COL6A6, TLR4, ANGPT1, PPP2R3C, PPP2R2A |
